# Magnetic ethylene-based periodic mesoporous organosilica supported palladium: An efficient and recoverable nanocatalyst for Suzuki reaction

**DOI:** 10.3389/fchem.2023.1112911

**Published:** 2023-02-02

**Authors:** Maryam Neysi, Dawood Elhamifar

**Affiliations:** Department of Chemistry, Yasouj University, Yasuj, Iran

**Keywords:** core-shell nanostructure, periodic nanoporous organosilica, Schiff-base, palladium, Suzuki reaction

## Abstract

In the present study, a novel magnetic ethylene-based periodic mesoporous organosilica supported Pd-Schiff base complex (Fe_3_O_4_@PMO/SB-Pd) was prepared, characterized and applied as a recoverable nanocatalyst for green synthesis of Suzuki products. Chemical composition, magnetic and thermal behavior, morphology and particle size of Fe_3_O_4_@PMO/SB-Pd were investigated by using FT-IR, TGA, EDX, VSM, PXRD, TEM and Scanning electron microscopy (SEM) analyses. The Fe_3_O_4_@PMO/SB-Pd nanocomposite was applied as an efficient nanocatalyst in the Suzuki reaction under ultrasonic conditions giving corresponding products in high yield. Some advantages of this study are simple purification of products, the use of water solvent, easy catalyst separation, short reaction time and high catalyst efficiency and recoverability.

## 1 Introduction

In recent decades, nanostructured catalysts have attracted a lot of attention due to their high-efficiency in organic reactions. Although nanocatalysts have a wide range of advantages including controllable size, biocompatibility and high efficiency for practical applications, however, their separation and reconstruction are often fraught with limitations and difficulties (Wei et al., 2012; Gawande et al., 2013; Gawande et al., 2015; Chen et al., 2021). The introduction of magnetic iron oxide nanoparticles suffer some of these problems and has led to the discovery of important criteria for the design of many novel and modern catalytic processes (Gawande et al., 2013; Kong et al., 2013; Garkoti et al., 2017). Especially, the use of Fe_3_O_4_ MNPs in the catalytic industry, which is based on principles of green chemistry, is very attractive in this matter. The unique properties of magnetic NPs such as biocompatibility and easy magnetic separation, has led other sciences such as chemistry, physics, pharmacy and medicine to pay particular attention to these particles. Therefore, the use of magnetic nanocatalysts not only saves time but also prevents problems such as catalyst degradation, catalyst oxidation and the preparation of organic waste (Gawande et al., 2015; Mirhosseini-Eshkevari et al., 2015; Zhang et al., 2017; Karimi et al., 2018; Kargar et al., 2020; Mousavi et al., 2021). Also, magnetite nanoparticles have wide applications in the drug delivery, cancer treatment, purification of water contaminated with heavy metals and radioactive materials, magnetic resonance imaging, etc. However, the use of iron oxide magnetic nanoparticles suffers from problems such as aggregation and oxidation, which has limited their range of application. Surface modification of iron oxide MNPs is a practical technique to prevent the aggregation and oxidation of these NPs that is achieved through the use of noble metals, metal oxides, silica and organic polymers (Neysi et al., 2019; Neysi et al., 2020; Li et al., 2021; Tang et al., 2021). Among different species, silica is the most common shell for the modification of the surface of magnetite NPs. On the other hand, periodic mesoporous organosilicas (PMOs) are a desirable class of organic-inorganic composite materials that have emerged as an ideal shell for MNPs due to their excellent properties such as high surface area, high lipophilicity and high thermal and mechanical stability (Liu, 2017; Zhao et al., 2017; Zhou et al., 2017; Elhamifar et al., 2018; Norouzi et al., 2018; Norouzi et al., 2019; Liu et al., 2022). Some of recently reported in this matter are Fe_3_O_4_@SiO_2_@PMO (Dai et al., 2017), Fe_3_O_4_ @SiO_2_@Am-PMO(Norouzi and Elhamifar, 2021), Fe_3_O_4_@RF@void@ PMO(IL)/Cu (Shaker and Elhamifar, 2021) and Fe_3_O_4_@MePMO-IL/Pd (Shaker and Elhamifar, 2020b).

In recent decades, Schiff-base ligands have attracted a lot of attention in the chemical and materials sciences due to their easy synthesis, easy complexation with the most of transition metal ions, good solubility and high catalytic properties. Moreover, Schiff-base ligand is considered as a linker between the catalytically active center and the solid materials to increase the catalytic activity (Ghorbani-Choghamarani et al., 2015; Zhao et al., 2018; Amirmahani et al., 2020; Zhou et al., 2020; Lashkari et al., 2021; Mazraati et al., 2021). Some of recently developed reports in this matter are Fe_3_O_4_@MCM-41-SB/Pd (Shaker and Elhamifar, 2020a), Fe_3_O_4_@BOS@SB/In (Mirbagheri and Elhamifar, 2019), Cu/SB-Fe_3_O_4_ (Elhamifar et al., 2017) and BPMO@ ISB/Mn (II) (Norouzi and Elhamifar, 2019).

The Suzuki reaction is an example of Pd-catalyzed cross-coupling processes where the coupling species are an aryl-boronic acid and an aryl or vinyl halide. The Suzuki products are widely used in the pharmaceutical industry, natural and pharmaceutical compounds, conductive polymers, sensors and dyes. Therefore, in recent years many researchers have studied and evaluated the optimization of this reaction using efficient catalytic systems (Dong et al., 2021; Favalli et al., 2021; Kempasiddaiah et al., 2021; Kim et al., 2021). Some of recently reported catalysts in this matter are Fe_3_O_4_@SiO_2_@NHC@Pd-MNPs (Akkoç et al., 2021), Fe_3_O_4_/Pd (Veisi et al., 2021), Fe_3_O_4_@SiO_2_/glucosamine-Pd (Eslahi et al., 2021), SiO_2_-NH_2_@Pd (dpa)Cl_2_ (Aghahosseini et al., 2021), Fe_3_O_4_/SiO_2_ -NH_2_@CS/Pd (Veisi et al., 2020), Fe_3_O_4_@MCM-41-SB/Pd (Shaker and Elhamifar, 2020a), PEt@ IL/Pd (Kargar and Elhamifar, 2020), GO-N_2_S_2_/Pd (Zarnegaryan and Elhamifar, 2020) and GO–SB/Pd (Zarnegaryan et al., 2019). In view of the above, in the present work, for the first time, a novel Fe_3_O_4_@Et-PMO supported Pd-Schiff base complex is prepared, characterized and its catalytic application is studied in the Suzuki reaction. Importantly, the present catalytic system has the advantages of magnetic Fe_3_O_4_ NPs, mesoporous materials and heterogeneous catalysts in the same time.

## 2 Experimental section

### 2.1 Production of Fe_3_O_4_@PMO

At first, Fe_3_O_4_ NPs were produced according to our previous reports (Neysi et al., 2019). Then, 0.5 g of these NPs were dispersed in a solution of H_2_O (80 mL) and EtOH (60 mL) at RT. Then, ammonia solution (25% wt, 10 mL) and cetyltrimethylammonium bromide (CTAB) (0.7 g) were added while stirring at the same temperature for 1 h. Next, a mixture of tetraethoxysilane (TEOS, 0.4 mL) and 1,2-bis(triethoxysilyl)ethane (BTEE, 0.7 mL) were dropwise added while stirring at the previous conditions for 1.5 h. After that, the obtained mixture was heated statically at 100°C for 48 h. The resulted magnetic Fe_3_O_4_@PMO was washed by using EtOH and H_2_O and dried. The removal of CTAB was achieved by using acidic hot EtOH.

### 2.2 Preparation of Fe_3_O_4_@PMO/SB

In order to preparation of Fe_3_O_4_@PMO/Pr-NH_2_, 0.5 g of Fe_3_O_4_@PMO was dispersed in toluene (20 mL) at RT. After adding 3-aminopropyltrimethoxysilane (0.5 mmol), the mixture was refluxed for 24 h. The product was separated using a magnet, dried and called Fe_3_O_4_@PMO/Pr-NH_2_. In the next step, 0.5 g of Fe_3_O_4_@PMO/Pr-NH_2_ was dispersed in toluene (20 mL) at RT. After adding 1.5 mmol of furfural, the resulted combination was refluxed for 24 h. The Fe_3_O_4_@PMO/SB was resulted after magnetic separation, washing and drying of the product.

### 2.3 Preparation of Fe_3_O_4_@PMO/SB-Pd

For this, 0.5 g of Fe_3_O_4_@PMO/SB was ultrasonically dispersed in DMSO (20 mL) for 20 min. Then, Pd(OAc)_2_.4H_2_O (0.75 mmol) was added while stirring at RT for 24 h. The Fe_3_O_4_@PMO/SB-Pd was resulted after magnetic separation, washing and drying of the product.

### 2.4 Suzuki reaction using Fe_3_O_4_@PMO/SB-Pd

For this purpose, 0.08 mol% of Fe_3_O_4_@PMO/SB-Pd, Ar-X (1 mmol), ArB(OH)_2_ (1.5 mmol), and K_2_CO_3_ (2 mmol) were added to H_2_O (10 mL) while ultrasonically stirring at 50°C. The reaction progress was monitored by using TLC. After completion of the reaction, ethyl acetate (10 mL) and H_2_O (5 mL) were added in the reaction mixture and the catalyst was magnetically separated. After decantation, the EtOAc phase was separated and dried over Na_2_SO_4_. The pure products were obtained after solvent evaporation or by isolation of the residue using column chromatography on silica.

## 3 Results and discussion

**FIGURE 1:**
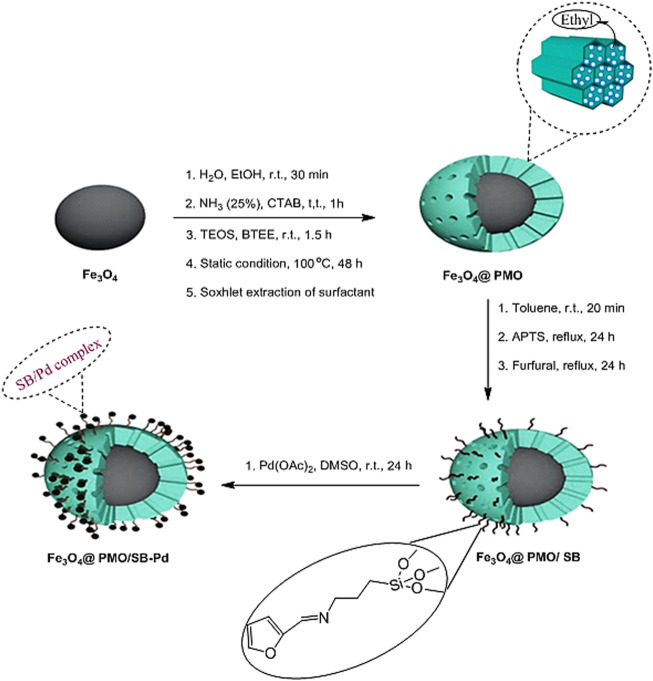
Production of Fe_3_O_4_@PMO/SB-Pd.

The synthesis method for the Fe_3_O_4_@PMO/SB-Pd is shown in Figure 1. For this, at first magnetite NPs were coated with periodic mesoporous organosilica shell *via* CTAB-directed hydrolysis and co-condensation of TEOS and BTEE. After CTAB removal, the obtained Fe_3_O_4_@PMO was modified with Schiff-base groups to deliver Fe_3_O_4_@PMO/SB. The Fe_3_O_4_@PMO/SB nanocomposite was finally treated with Pd(OAc)_2_.4H_2_O to give Fe_3_O_4_@PMO/SB-Pd catalyst. The chemical and structural properties of the Fe_3_O_4_@PMO/SB-Pd catalyst were investigated using FT-IR, VSM, SEM, EDX, TEM and PXRD analyses.

**FIGURE 2:**
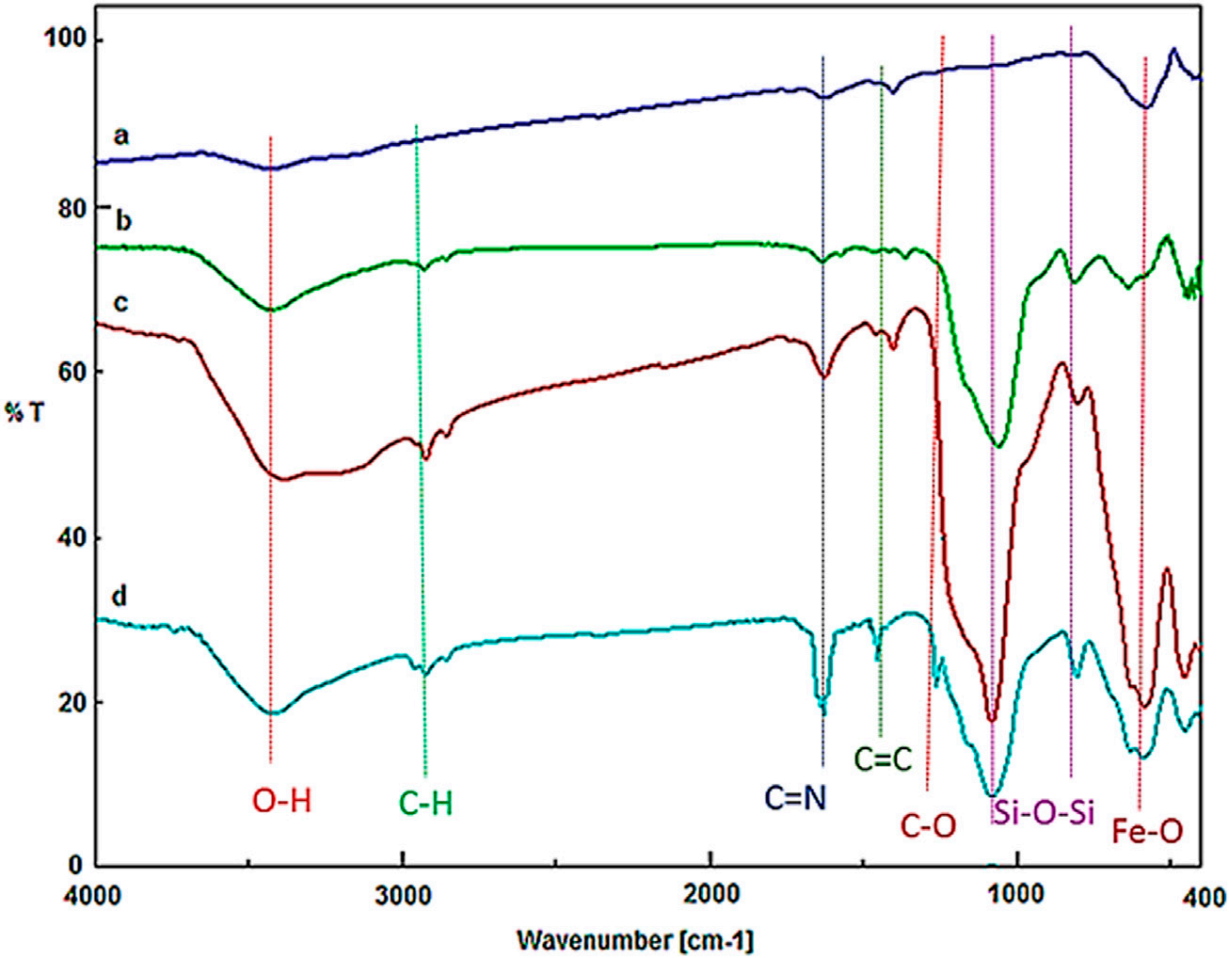
FT-IR of **(A)** Fe_3_O_4_, **(B)** Fe_3_O_4_@PMO, **(C)** Fe_3_O_4_@PMO/Pr-NH_2_ and **(D)** Fe_3_O_4_@PMO/SB-Pd.

The FT-IR spectra of Fe_3_O_4_, Fe_3_O_4_@PMO, Fe_3_O_4_@PMO/Pr-NH_2_ and Fe_3_O_4_@PMO/SB-Pd are depicted in Figure 2. The signals at 588 and 3,300–3,450 cm^−1^ are, respectively, assigned to Fe-O and O-H bonds. Also, for Fe_3_O_4_@PMO, Fe_3_O_4_@PMO/Pr-NH_2_ and Fe_3_O_4_@PMO/SB-Pd, the peaks observed at 823 and 1076 cm^−1^ are related to the Si-O-Si bonds. Moreover, the peaks at 2,921 and 2,853 cm^−1^ are for C-H bonds of Et-PMO and propyl groups. Interestingly, for the Fe_3_O_4_@PMO/SB-Pd nanocomposite, the peaks at 1100, 1428 and 1623 cm^-1^ are, respectively, for the C-O, C=C and C=N bonds of the SB complex. These results confirm the successful formation and high stability of Et-PMO and SB groups into/onto the material framework.

**FIGURE 3:**
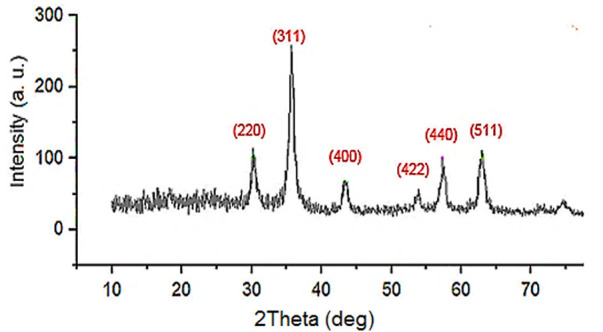
Wide-angle PXRD of Fe_3_O_4_@PMO/SB-Pd.

The wide-angle PXRD of the Fe_3_O_4_@PMO/SB-Pd nanocatalyst is illustrated in Figure 3. This analysis revealed the signals at 2Ɵ = 30.3, 35.7, 43.4, 53.8, 57.7 and 63.0 degrees that are, respectively, due to the reflections of 220, 311, 400, 422, 511 and 440, confirming the crystalline structure of MNPs is stable and not changed during preparation of Fe_3_O_4_@PMO/SB-Pd (Figure 3).

**FIGURE 4:**
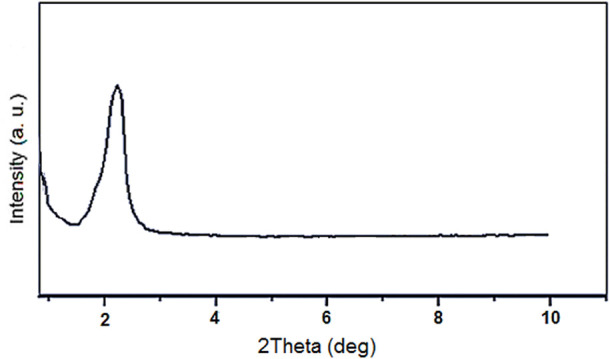
Low-angle PXRD of Fe_3_O_4_@PMO/SB-Pd.

The low-angle PXRD analysis of Fe_3_O_4_@PMO/SB-Pd nanocatalyst showed a peak at 2.2° that is attributed to the mesoporous structure of the PMO shell (Figure 4).

**FIGURE 5:**
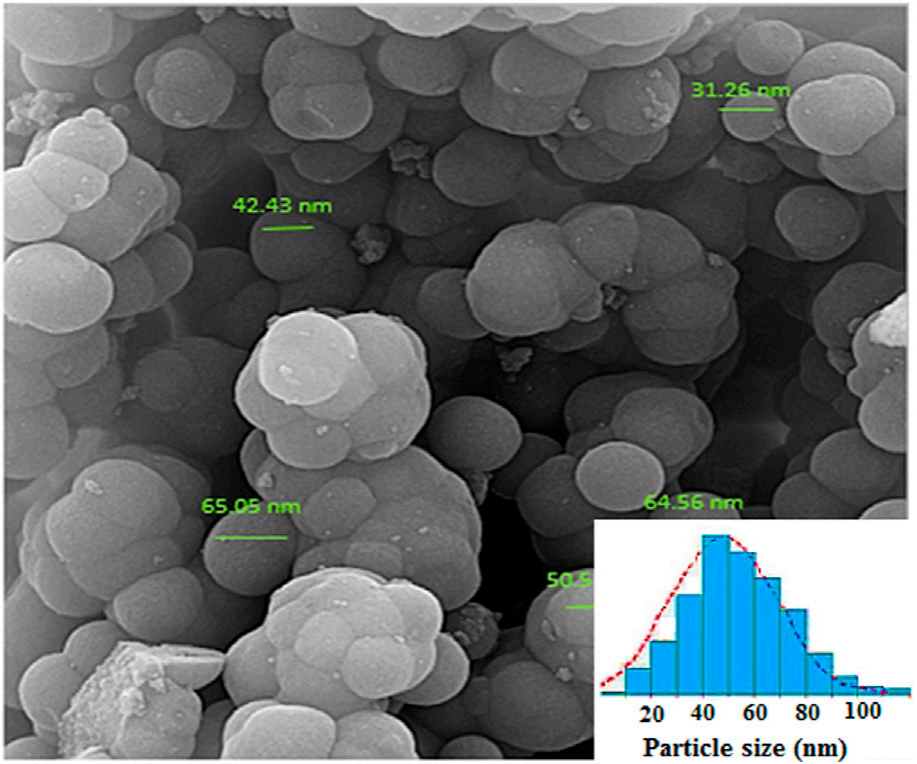
SEM of Fe_3_O_4_@PMO/SB-Pd.

Scanning electron microscopy (SEM) analysis of Fe_3_O_4_@PMO/SB-Pd nanocatalyst revealed spherical particles for the designed catalyst (Figure 5). The average particle size of Fe_3_O_4_@PMO/SB-Pd NPs was about 50 nm according to the particle size distribution histogram.

**FIGURE 6:**
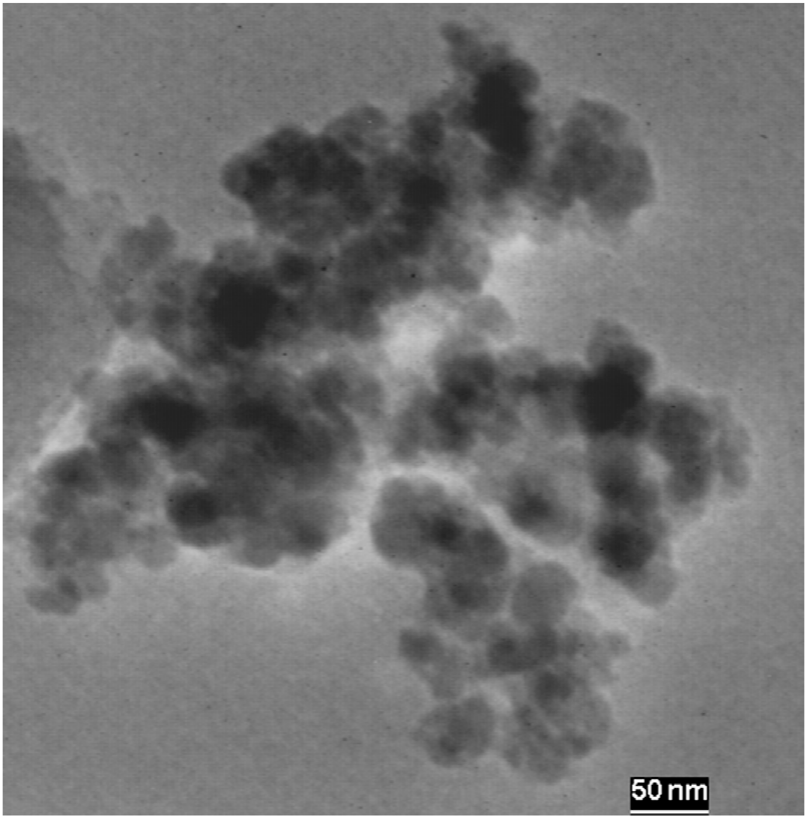
TEM of Fe_3_O_4_@PMO/SB-Pd.

The transmission electron microscopy image of the Fe_3_O_4_@PMO/SB-Pd nanocatalyst demonstrated that the Fe_3_O_4_@PMO/SB-Pd nanocatalyst has a core-shell structure with a black core (magnetite NP) and a gray shell (mesoporous layer) (Figure 6).

**FIGURE 7:**
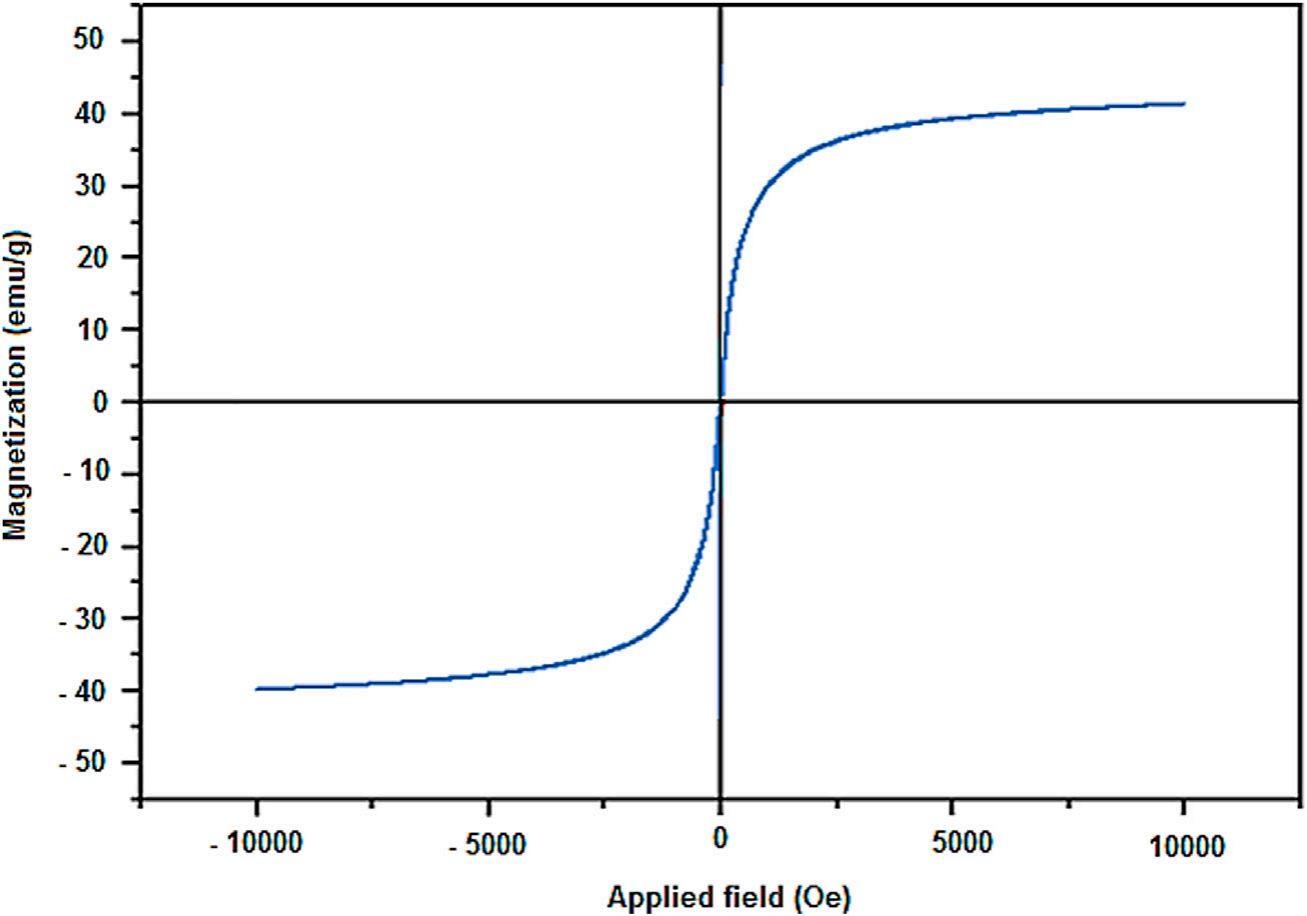
VSM analysis of Fe_3_O_4_@PMO/SB-Pd.

The magnetic properties Fe_3_O_4_@PMO/SB-Pd were determined using vibrating sample magnetometer analysis (VSM). This analysis illustrated that the magnetic saturation of the Fe_3_O_4_@PMO/SB-Pd nanocatalyst is about 40 emu g^−1^, which is a confirmation of its high magnetic property (Figure 7).

**FIGURE 8:**
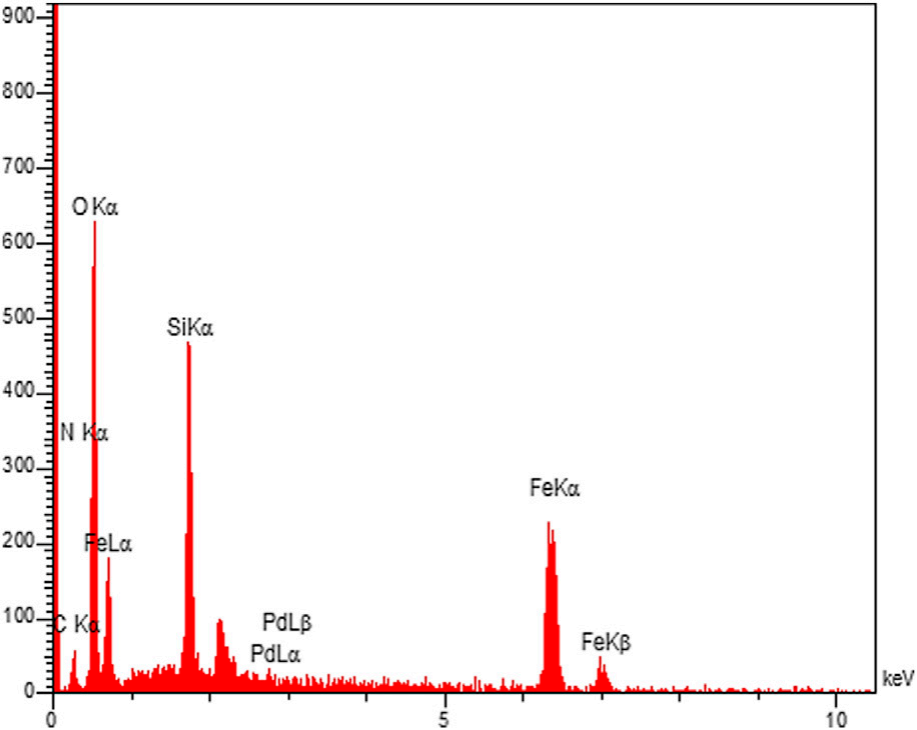
EDX of Fe_3_O_4_@PMO/SB-Pd.

The presence of the desired elements in the Fe_3_O_4_@PMO/SB-Pd nanocatalyst was confirmed using EDX analysis. This showed the signals of the elements of Fe, Si, C, N, Pd and O in the catalyst, which confirms the high stability of the expected organic and inorganic groups onto/into material framework (Figure 8).

**FIGURE 9:**
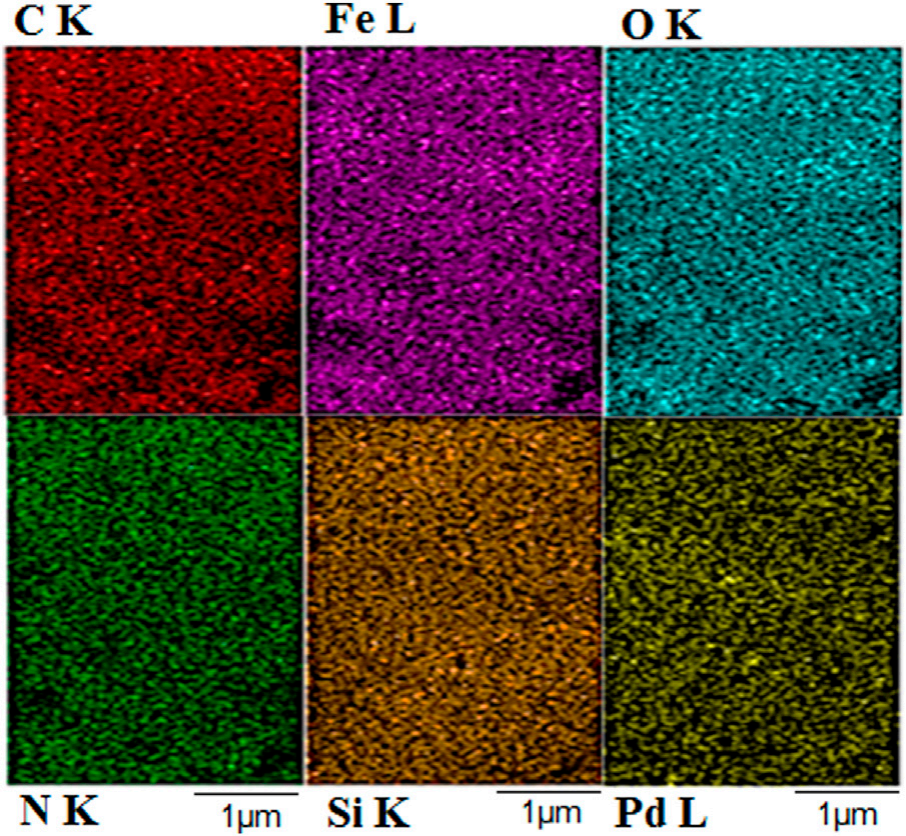
EDX mapping of Fe_3_O_4_@PMO/SB-Pd.

The elemental mapping analysis also indicated the uniform distribution of all elements in the material framework (Figure 9).

**FIGURE 10:**
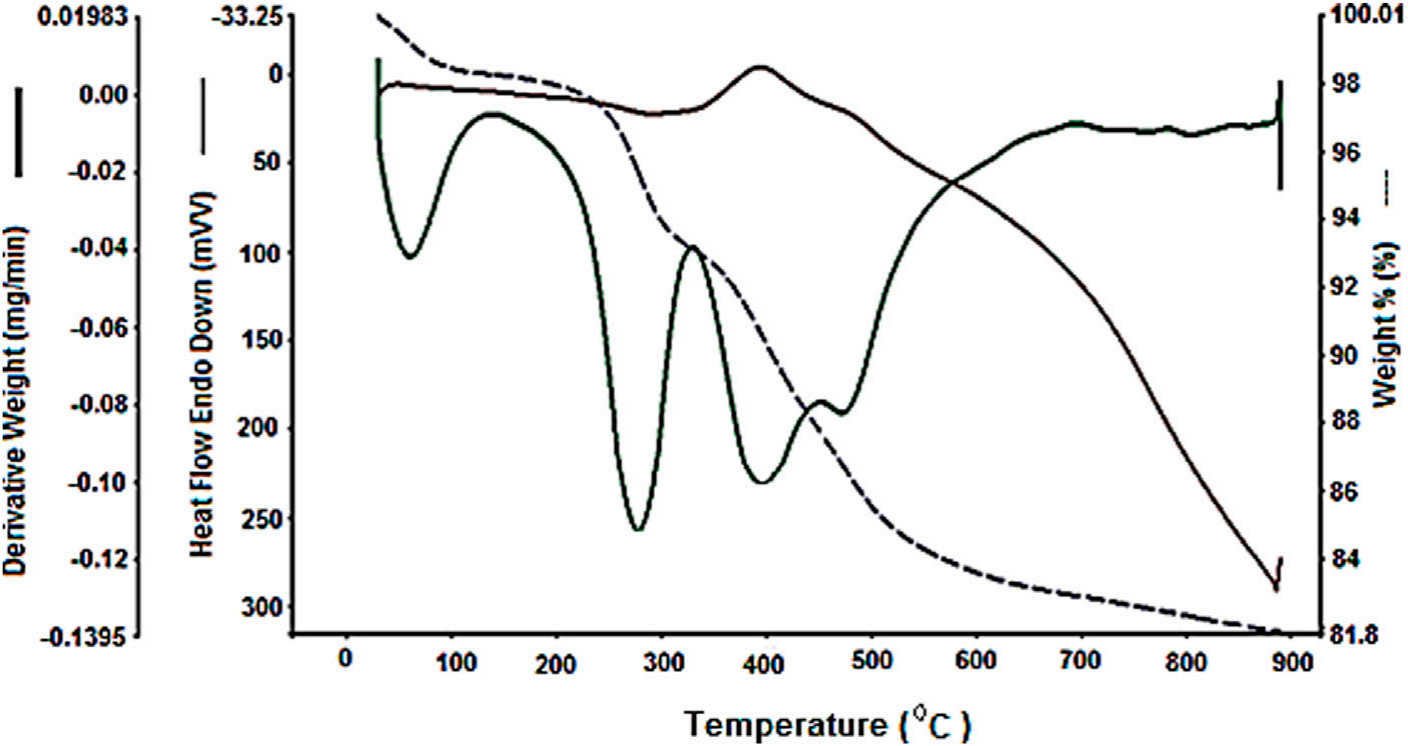
TGA of Fe_3_O_4_@PMO/SB-Pd.

Finally, TGA was done to study the stability of Fe_3_O_4_@PMO/SB-Pd. The first weight loss at 25°C–150°C is attributed to removal of water and organic solvents remaining in the synthesis process. The next weight loss in the range of 200°C–300°C is related to the decomposition of the p123 surfactant, which remains after the extraction process. The main weight loss, which appears at 301°C–850°C is due to the decomposition and removal of incorporated/immobilized organic functional groups (ethylene and Schiff-base) onto/into the structure of Fe_3_O_4_@PMO/SB-Pd nanocomposite (Figure 10).

Subsequently, the catalytic activity of Fe_3_O_4_@PMO/SB-Pd in the Suzuki reaction was investigated. The condensation between iodobenzene and PhB(OH)_2_ was selected to achieve the best conditions (Table 1). At first, the effect of catalyst loading was investigated. As shown, the reaction yield is increased with the increasing amount of catalyst in which the highest product yield is obtained using 0.08 mol% of Fe_3_O_4_@PMO/SB-Pd (Table 1, entries 1–3). Also, the temperature effect study showed that at 50°C under ultrasonic conditions the best result is obtained (Table 1, entries 2, 4–6). The study also confirmed that the result of ultrasonic bath is much better than oil bath under the same conditions (Table 1, entry 2 versus 7). Various solvents were also examined (Table 1, entries 2, 8–10), which, water as environmentally-friendly solvent gave the highest yield. Among different bases of NaOAc, NaOH, K_2_CO_3_ Et_3_N and base-free media, K_2_CO_3_ was the best (Table 1, entry 2 vs*.* entries 11–14). Accordingly, the use of 0.08 mol% of Fe_3_O_4_@PMO/SB-Pd and K_2_CO_3_ in H_2_O at 50°C under ultrasonic irradiations were chosen as optimum conditions. In the next step, the Suzuki reaction was performed using Pd-free Fe_3_O_4_@PMO and Fe_3_O_4_@PMO/SB nanomaterials under the same conditions as the Fe_3_O_4_@PMO/SB-Pd nanocatalyst. Interestingly, in the latter cases no product was obtained confirming the process is catalyzed by supported Pd species (Table 1, entries 15, 16). It is well-known that in the Pd-catalyzed Suzuki reaction, the active catalytic species are Pd (0). In the present study, although the oxidation state of supported Pd is (II), however, during the reaction conditions this converts to active Pd (0) to successfully catalyze the Suzuki process (Karimi et al., 2010; Karimi et al., 2011; Elhamifar et al., 2013).

**TABLE 1:** The effect of solvent, temperature and base in the Suzuki reaction.

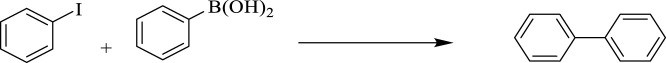						
Entry	Solvent	Base	Cat. (mol%)	T (°C)	Time (min)	Yield (%)
1	H_2_O	K_2_CO_3_	0.04	50	30	83
2^a^	H_2_O	K_2_CO_3_	**0.08**	**50**	**30**	**96**
3	H_2_O	K_2_CO_3_	0.16	50	30	96
4	H_2_O	K_2_CO_3_	0.08	r.t.	30	Trace
5	H_2_O	K_2_CO_3_	0.08	40	30	60
6	H_2_O	K_2_CO_3_	0.08	65	30	96
7^b^	H_2_O	K_2_CO_3_	0.08	50	30	75
8	EtOH	K_2_CO_3_	0.08	50	30	82
9	Toluene	K_2_CO_3_	0.08	50	30	55
10^c^	H_2_O/EtOH	K_2_CO_3_	0.08	50	30	88
11	H_2_O	NaOAc	0.08	50	30	78
12	H_2_O	NEt_3_	0.08	50	30	67
13	H_2_O	NaOH	0.08	50	30	60
14	H_2_O	Base-free	0.08	50	60	Trace
15^d^	H_2_O	K_2_CO_3_	0.004 g	50	60	—
16^e^	H_2_O	K_2_CO_3_	0.004 g	50	60	—

^a^ Optimum conditions.

^b^ The reaction was performed in an oil bath.

^c^ EtOH:H_2_O (1:1).

^d^ Fe_3_O_4_@PMO/SB was used as catalyst.

^e^ Fe_3_O_4_@PMO was used as catalyst.

After optimization, the efficiency of Fe_3_O_4_@PMO/SB-Pd nanocatalyst was investigated in the synthesis of biphenyl products *via* Suzuki reaction. As shown in Table 2, all arylhalides including aryl-iodide, bromide and chloride, with different substituents, have been used as substrate giving corresponding coupling adducts in good to high yield and selectivity. These results show the high efficiency of Fe_3_O_4_@PMO/SB-Pd for synthesis a wide-range of Suzuki products.

**TABLE 2:** Suzuki reaction in the presence of Fe_3_O_4_@PMO/SB-Pd.

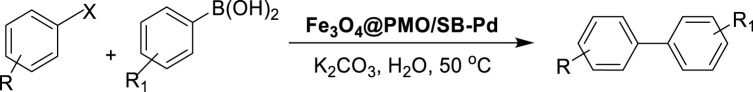							
Entry	Ar-X	ArB(OH)_2_	Time (min)	Yield^a^ (%)	TON^b^	TOF^c^	Found M.P. (°C)
1	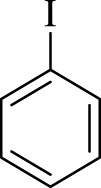	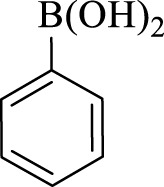	30	96	1200	2400	68–70
2	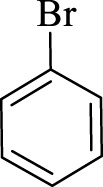	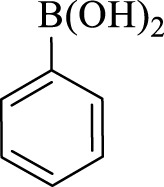	50	95	1187.5	1430.7	68–70
3	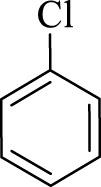	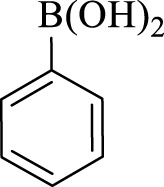	75	85	1062.5	850	68–70
4	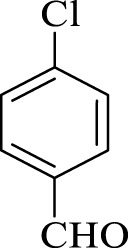	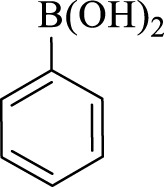	60	88	1100	1100	57–59
5	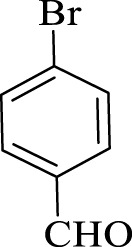	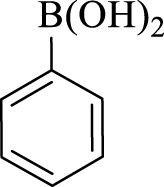	40	90	1125	1704.5	57–59
6	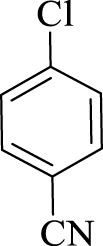	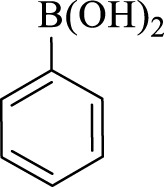	75	90	1125	900	48–50
7	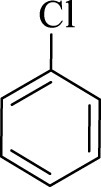	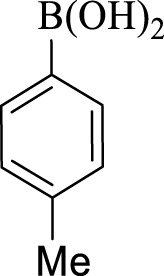	75	86	1075	860	47–49
8	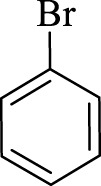	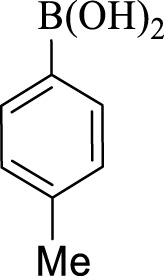	50	93	1162.5	1400.6	47–49

^a^ Isolated yield.

^b^ Turnover number [defined as yield (%)/cat. (mol%)].

^c^ Turnover frequency [defined as TON/reaction time (h)].

**FIGURE 11:**
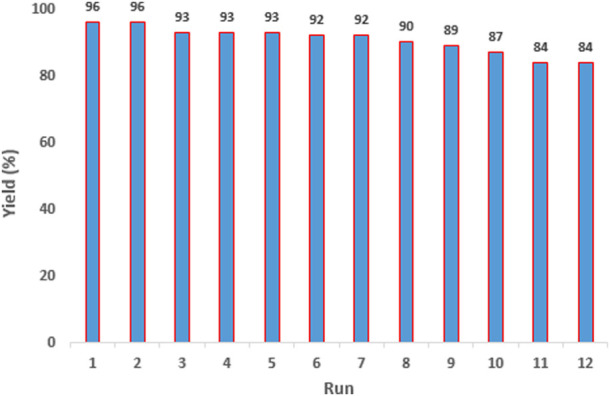
The reusability and recoverability of Fe_3_O_4_@PMO/SB-Pd.

The reusability and recoverability of the Fe_3_O_4_@PMO/SB-Pd nanocatalyst were investigated under optimal conditions in the Suzuki reaction between PhB(OH)_2_ and PhI. For this, after completion of the reaction, the catalyst was recovered and reused in the next run. The results showed that the nanocatalyst can be reused and recovered at least eleven times with only a slight decrease in the product yield after each run (Figure 11).

**FIGURE 12:**
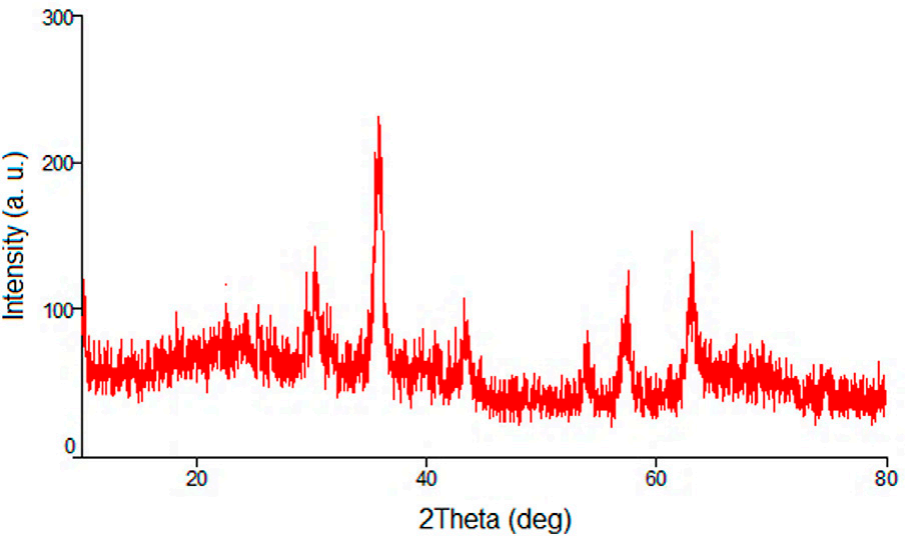
The PXRD analysis of the recovered nanocatalyst.

In the next, the PXRD analysis of the recovered catalyst was performed to study its chemical stability under applied conditions (Figure 12). As shown, the pattern of this analysis is the same as PXRD of the fresh catalyst confirming high stability of the crystalline structure of the iron oxide NPs during the reaction conditions.

The leaching test of Fe_3_O_4_@PMO/SB-Pd was also investigated under optimal conditions in the Suzuki reaction between PhB(OH)_2_ and PhI. After about 50% progress, the reaction was stopped and the catalyst was magnetically removed. The reaction of the residue was monitored and the result revealed no progress after 2 h. This indicates that the Fe_3_O_4_@PMO/SB-Pd nanocatalyst acts heterogeneously and also confirms that the palladium moieties are well stabilized on the material.

Finally, the catalytic activity of Fe_3_O_4_@PMO/SB-Pd nanocatalyst was compared with various catalysts that have recently been used in the Suzuki reaction. As shown in Table 3, the new catalyst possesses better performance than others in terms of temperature, reaction rate and recyclability.

**TABLE 3:** Comparison the efficiency and recoverability of Fe_3_O_4_@PMO/SB-Pd with previous catalysts.

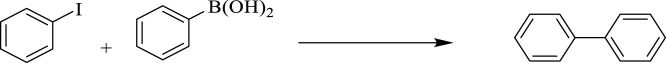				
Catalyst	Conditions	Time	Recovery times	References
Pd-γ-Fe_2_O_3_	Cat. 0.5 mol%, 60°C aceton/H_2_O, K_3_PO_4_	4 h	3	Paul et al. (2018)
Fe_3_O_4_@SiO_2_@mSiO_2_-Pd	Cat. 0.075 mol%, 80°C, isopropyl alcohol, K_2_CO_3_	6 h	4	Sharma et al. (2016)
KCC-1-NH_2_/Pd^a^	Cat. 0.5 mol%, 100°C, K_3_PO_4_, EtOH/H_2_O	4 h	7	Fihri et al. (2012)
Fe_3_O_4_@SiO_2_/isoniazide/Pd	Cat. 0.2 mol%, 50°C, EtOH-H_2_O, K_2_CO_3_	30 min	7	Heidari et al. (2017)
Pd@MNP	Cat. 0.2 mol%, 60°C, EtOH/H_2_O, K_2_CO_3_	4 h	5	Zhang et al. (2013)
Pd (L_8_)_2_	Cat. 0.75 mol%, RT, EtOH/H_2_O, K_2_CO_3_	24 h	5	Neshat et al. (2021)
Starch-Fe_3_O_4_@IL-TZ-Pd^b^	Cat. 0.05 mol%, RT, EtOH/H_2_O, K_2_CO_3_	4 h	10	Gholinejad et al. (2021)
Pd@C-dots@Fe_3_O_4_ ^c^	Cat. 0.22 mol%, RT, EtOH/H_2_O, K_2_CO_3_	2 h	8	Gholinejad et al. (2016)
Fe_3_O_4_@PMO/SB-Pd	Cat. 0.08 mol%, 50°C, H_2_O, K_2_CO_3_	30 min	11	This work

^a^ KCC-1, fibrous nano-silica.

^b^ TZ, triazole.

^c^ C-dots, carbon quantum nanodots.

## Conclusion

In summary, the Fe_3_O_4_@PMO/SB-Pd catalyst was successfully prepared and employed in the Suzuki reaction. The FT-IR, EDX and TGA analyses showed high stability of organic and palladium moieties on material framework. The VSM and XRD analyses proved high magnetic properties of the designed catalyst. The SEM and TEM analyses also showed a spherical morphology for Fe_3_O_4_@PMO/SB-Pd. This nanocatalyst was effectively employed in the Suzuki reaction giving coupling products in high yield. Fe_3_O_4_@PMO/SB-Pd was also recovered and re-employed several times with no significant reduction in its performance.

## Data Availability

The original contributions presented in the study are included in the article/Supplementary Material, further inquiries can be directed to the corresponding author.
